# Regularized multi-trait multi-locus linear mixed models for genome-wide association studies and genomic selection in crops

**DOI:** 10.1186/s12859-023-05519-2

**Published:** 2023-10-26

**Authors:** Aurélie C. Lozano, Hantian Ding, Naoki Abe, Alexander E. Lipka

**Affiliations:** 1 IBM Research AI, IBM T.J. Watson Reseach Center, Yorktown Heights, USA; 2 University of Pennsylvania, Philadelphia, USA; 3 Department of Crop Sciences, University of Illinois, Urbana-Champaign, USA

**Keywords:** Multi-trait multi-locus linear mixed model, GWAS and genomic selection in plants, Regularization

## Abstract

**Background:**

We consider two key problems in genomics involving multiple traits: multi-trait genome wide association studies (GWAS), where the goal is to detect genetic variants associated with the traits; and multi-trait genomic selection (GS), where the emphasis is on accurately predicting trait values. Multi-trait linear mixed models build on the linear mixed model to jointly model multiple traits. Existing estimation methods, however, are limited to the joint analysis of a small number of genotypes; in fact, most approaches consider one SNP at a time. Estimating multi-dimensional genetic and environment effects also results in considerable computational burden. Efficient approaches that incorporate regularization into multi-trait linear models (no random effects) have been recently proposed to identify genomic loci associated with multiple traits (Yu et al. in Multitask learning using task clustering with applications to predictive modeling and GWAS of plant varieties. arXiv:1710.01788, 2017; Yu et al in Front Big Data 2:27, 2019), but these ignore population structure and familial relatedness (Yu et al in Nat Genet 38:203–208, 2006).

**Results:**

This work addresses this gap by proposing a novel class of regularized multi-trait linear *mixed* models along with scalable approaches for estimation in the presence of high-dimensional genotypes and a large number of traits. We evaluate the effectiveness of the proposed methods using datasets in maize and sorghum diversity panels, and demonstrate benefits in both achieving high prediction accuracy in GS and in identifying relevant marker-trait associations.

**Conclusions:**

The proposed regularized multivariate linear mixed models are relevant for both GWAS and GS. We hope that they will facilitate agronomy-related research in plant biology and crop breeding endeavors.

**Supplementary Information:**

The online version contains supplementary material available at 10.1186/s12859-023-05519-2.

## Background

The effective use of DNA markers from high-throughput genomic data in breeding will be critical for increasing agronomic productivity to levels that will be able to sustain the population by 2050 [1]. In particular, use of these markers in statistical analyses will facilitate gene identification via genome-wide association studies (GWAS). Ever since its first documented use in [2], GWAS has successfully identified marker-trait associations with moderate- to large-effect sizes associated with various agronomical traits.

The type of data sets most widely used for plant GWAS is the diversity panel [reviewed in 3]. The individuals in diversity panels typically cluster into sub-populations, and the degree of familial relatedness among these individuals differ [as seen in 4, 5]. If these two sources of variability are not accounted for in the statistical analysis conducted in GWAS, then the most statistically significant marker-trait associations are likely to arise from population structure and/or familial relatedness. These spurious associations obfuscate the identification of loci that are in linkage disequilibrium with causal mutations underlying the tested trait. One of the most widely-used GWAS approaches to account for spurious associations is the linear mixed model (LMM) [6], which includes fixed effect covariates to account for sub-population structure and random effects to account for the genetic relatedness between individuals. The LMM is arguably the most widely-used model in plant GWAS, and its proven effectiveness in accounting for false positive marker-trait associations have kept it at the forefront of quantitative genetics research for over 15 years. Various iterations of the LMM have been proposed [7–10], and today the most widely used version of the LMM in plant GWAS is the single-trait LMM fitted to each SNP across a species genome.

Complementing the availability of high-throughput genotypic data, high-throughput phenotypic data are increasingly available and enable genome-wide analysis of multiple traits [11]. As such, multivariate models are starting to become more widely used for GWAS because they include multiple traits and can utilize the covariance between them to quantify marker-trait associations with greater precision. For plant GWAS, the multivariate analogue of the LMM is the multi-trait linear mixed model [mtLMM; 12, 13]. In addition to including random effects that capture the relatedness between individuals and covariance between multiple traits, the mtLMM is capable of capturing covariance between environments and residual error. While there has been recent work on scaling mtLMM estimation with respect to the number of traits [14], the most widely used versions of the mtLMM still test only one SNP at a time [e.g. see 14–16].

To enable the mtLMM to quantify the simultaneous contributions of multiple loci underlying a set of multivariate traits as accurately as possible, statistical approaches are needed that fit all genome-wide markers available into one model. There has been recent work in the machine learning community on approaches that incorporate regularization techniques to identify genomic loci associated with multiple phenotypic traits for the multivariate (multi-trait) linear model (mtLM). The mtLM differs from the mtLMM in that it only includes fixed SNP effects as the explanatory variables and no random effect covariates accounting for spurious marker-trait associations are considered. One key feature of all of these regularized approaches [e.g., 17–19] is that they all strive to provide biologically meaningful estimates of marker effects while addressing the inherent issue of the number of available markers (*p*) exceeding the number of individuals in the data set (*n*; i.e., the $p>> n$ issue). While these approaches are promising, there is a critical need to incorporate the approaches used in the mtLMM to account for spurious associations that typically arise in diversity panels. Otherwise, it might be that the regularized multi-locus mtLM approaches will not be able to identify genomic signals linked to the actual genes underlying the studied traits.

In this work, we propose a class of regularized mtLMMs with scalable optimization for model estimation. By incorporating regularization into the mtLMM, we are able to accommodate high-dimensional GWAS. We show that this regularization facilitates the identification of genomic regions with peak GWAS associations. We also show that the proposed class of mtLMMs is competitive for use in genomic selection (GS), where genome-wide markers are used to estimate trait breeding values in a set of breeding material [20]. To overcome the computational challenges faced by existing methods for mtLMMs and realize efficient and scalable estimation, we leverage recent advances in proximal-based optimization methods. As special cases of our formulation, we consider mtLMMs with sparsity-inducing regularization to reflect the thesis that only a subset of markers available from high-throughput genotypic data are predictive of or associated to the traits under consideration [21]. We also consider a ‘convex clustering’ regularization to leverage model relatedness across traits, which is pertinent in settings in which groups of traits are either strongly correlated or potentially also controlled by pleiotropic genes, i.e. genes that have effects on multiple traits. We evaluate our approach in two crop diversity panels, and demonstrate the benefits for both GWAS and GS, respectively in terms of peak association identification and prediction accuracy. Although we specifically focus on crops for this work, these approaches are applicable to any species.

## Materials and methods

### Multi-trait linear mixed model (mtLMM)

Suppose that we observe *q* traits and *p* covariates (e.g. SNPs) for *n* individuals. We consider a multi-trait linear mixed model to relate the multiple phenotypes and genotypes:

1 $$\begin{aligned} \varvec{Y} = \varvec{X} \varvec{B} + \varvec{G} + \varvec{E} \end{aligned}$$

where $\varvec{Y}$ is a $n \times q$ matrix of traits, $\varvec{X}$ is a $n \times p$ covariate matrix for the fixed effects, including the SNPs being tested, $\varvec{B}$ is a $p \times q$ matrix of effect sizes, $\varvec{G}$ is a $n \times q$ matrix representing the genetic background component, and $\varvec{E}$ is a $n \times q$ matrix representing the noise component due to environment and error. The random effects $\varvec{G}$ and $\varvec{E}$ follow a matrix-variate normal distribution:

2 $$\begin{aligned}&\varvec{G}\sim N_{n \times q}(\varvec{0},\varvec{C}_g,\varvec{K})\end{aligned}$$

3 $$\begin{aligned}&\varvec{E}\sim N_{n \times q}(\varvec{0},\varvec{C}_e,\varvec{I}_n), \end{aligned}$$

where ${{\textbf {K}}}$ is the $n \times n$ kinship matrix and $N_{n \times q}(\varvec{M},\varvec{\Psi },\varvec{\Phi })$ denotes the matrix-variate normal distribution with mean matrix $\varvec{M}$ and column and row covariance matrices $\varvec{\Psi }$ and $\varvec{\Phi },$ $\varvec{I}_n$ is the $n \times n$ identity matrix, $\varvec{C}_g$ and $\varvec{C}_e$ are respectively the genetic and environment covariance matrices.

The distribution of $\varvec{Y}$ can be written concisely as

$$\begin{aligned} \varvec{Y}=N_{n \times q}(\varvec{X} \varvec{B},\varvec{C}_g,\varvec{K})+N_{n \times q}(0,\varvec{C}_e,\varvec{I}_n) \end{aligned}$$

Using the $\textrm{vec}$ operator to vectorize a matrix, concatenating its columns, the distribution can also be represented using the multivariate normal distribution as

$$\begin{aligned} \textrm{vec}(\varvec{Y}) \sim N_{nq}(\textrm{vec}(\varvec{X} \varvec{B}),\varvec{C}_g \otimes \varvec{K} + \varvec{C}_e \otimes \varvec{I}_n) \end{aligned}$$

Here $\otimes$ is the Kronecker product and $N_{nq}$ is the usual multivariate normal distribution of dimension *nq*.

*mtLMM Estimation: Prior work and limitations.*

Prior work first estimates $\varvec{C}_g$ and $\varvec{C}_e$ from the null model (i.e. $\textrm{vec}(\varvec{Y}) \sim N_{nq}(0,\varvec{C}_g \otimes \varvec{K} + \varvec{C}_e \otimes \varvec{I}_n$) using Maximum Likelihood (ML) or Restricted Maximum Likelihood (REML) estimation. For this, efficient inference schemes have been proposed that exploit Kronecker identities for the eigen-decomposition of the covariance matrix. Then genetic variants (SNPs) are tested one by one, either by keeping the estimated $\varvec{C}_g$ and $\varvec{C}_e$ from the null model [15, 16, 22, 23] or by refitting for each variant tested [13]. The computational complexity of existing approaches to estimate $\varvec{C}_g$ and $\varvec{C}_e$ is acceptable when the number of traits is relatively small.

When *q* is larger, [14] estimates the variance components using *b* *s*-sized subsets of $\{1,\ldots ,q\}$, and the estimation for each of the *b* bootstrap subsets can be performed in parallel.

None of these approaches, however, are able to perform estimation for the complete mtLMM where the effects of all SNPs are considered simultaneously. In such a case *X* is high-dimensional, i.e., $p>>n$. Existing approaches only test one SNP at a time, and could only accommodate a few SNPs at best, since they rely on inverting matrices or on solving linear systems that are no longer invertible or well posed when *p* is larger than *n*. Even if this critical issue could be bypassed, the computational complexity of the fixed effect coefficient estimation step would be impractical [see [15, 16], for examples where the required computational complexity is respectively cubic and quadratic in the number of fixed effects *p*].

### Regularized multi-trait linear mixed model

To address the aforementioned issues, we propose a class of regularized mtLMMs that enables estimation in the high-dimensional setting where the number of fixed effects *p* can be significantly larger than the number of observations *n*. Regularization has been a very active topic in univariate and multivariate linear models. It has also been employed in univariate mixed effect models for variable selection [see [24, 25], and references therein]. Although an analogous multi-trait Bayesian approach has been investigated [26], regularization has not yet been leveraged in the multi-trait case. Note that [27] considers a regularized multivariate mixed model for longitudinal data, which cannot be applied to the present context as assumptions are made on the structure of the matrices in the model that reflect the longitudinal nature of the data. Moreover, a two-step procedure is used for estimation: first the random effect covariance matrices are estimated from individual univariate mixed models. The random effect parameters are then set to these estimated values, and then a basic penalized linear regression problem is used to solve for the fixed effect. Concurrently with our work, [28] proposed a Bayesian multi-trait mixed model framework specifically geared towards genomic prediction. Their framework, however, employs factors to model the joint effects of all predictors (fixed, random and residual) on multiple traits and is therefore not applicable to GWAS.

The framework proposed in this paper minimizes a class of penalized negative log-likelihood functions:

$$\begin{aligned}\underset{\varvec{B}, \varvec{C}_g, \varvec{C}_e}{\textrm{minimize}} -LL(\varvec{B},\varvec{C}_g,\varvec{C}_e) + {\mathcal {R}}(\varvec{B}),\end{aligned}$$

where ${\mathcal {R}}(\cdot )$ denotes a regularization function.

Examples of regularization functions include the following:

*Example 1: Variable Selection.*

Variable selection can be performed using

$$\begin{aligned}{\mathcal {R}}(\varvec{B}) = \lambda \sum _{i,j} |B_{ij}|,\end{aligned}$$

where $\lambda \ge 0$ is a regularization parameter. One can employ other penalties encouraging sparsity in the model coefficients such as SCAD [29].

*Example 2: Variable Selection and Trait-wise Clustering.*

In several applications it may make sense to assume that there is an underlying grouping of traits, where traits in the same group are impacted by a similar set of SNPs. To identify and leverage such a grouping, one can employ the convex clustering penalty as proposed in [17], adapted here for the multivariate linear model.

$$\begin{aligned}{\mathcal {R}} = \lambda \sum _{i,j} |B_{ij}| + \gamma \sum _{(j,j')\in E} c_{jj'} \Vert B_{:j}-B_{:j'}\Vert .\end{aligned}$$

The first component of the penalty encourages sparsity of the parameter matrix $\varvec{B}$. The second term encourages similarity of the parameters across pairs of traits. Coefficients $c_{jj'}$ are optional and are provided a-priori based on domain knowledge of which traits are more likely to exhibit similar associations. See [17] for more details.

An optimization procedure to efficiently estimate regularized mtLMMs is presented in Additional file 2: Appendix.

### Datasets

We evaluated the performance of regularized mtLMMs against alternate approaches by first analyzing real traits from two crop diversity panels. The first of these two datasets is the Goodman diversity panel, which consists of 281 maize (*Zea mays* L.) diverse lines [30]. We considered a subset of 215 lines that had no missing phenotypic information across all 20 tocochromanol grain traits that were evaluated in [31]. Thus the statistical models we considered included all of these 20 tocochromanol traits as the response variable, and were tested against a subset of 3,092 genome-wide markers described in [31]. We also used the kinship matrix from [31], which was calculated from the method of [32], in subsequent analyses to account for relatedness among the individuals.

The second dataset analyzed is a diversity panel of 320 sorghum (*Sorghum bicolor* L.) lines that have been previously described in [5] and [33]. We analyzed a total of six traits, specifically plant height (PH), preflag leaf height (PLH), preflag to flag (PTF), flag to apex (FTA), rachis length (RL), and branch length (BL) [as described in 5, 33]. For this analysis a total of 116, 128 genotyping-by-sequencing [GBS; 34] markers originally described in [35] were considered. Similar to the maize data, the kinship matrix from [36], which was calculated using the method of [32], was used to account for relatedness among the individuals.

### Evaluating the potential of regularized mtLMMs for genomic selection

To assess the ability of our proposed models to accurately predict genomic estimated breeding values (GEBVs), a total of six different models were fitted to the maize and sorghum data. Such an evaluation is a critical first step for determining the potential application of these models to GS in plant breeding. The specific models we evaluated include the multitrait linear mixed model with L1 regularization (mtLMM-L1), the multitrait linear model with L1 regularization (mtLM-L1), the multitrait mixed linear model with convex clustering regularization (mtLMM-clust), and the multitrait linear model with convex clustering regularization (mtLM-clust). Each of these models used all available traits within a given data set as the vector of response variables and all available genome-wide markers as explanatory variables. As an additional comparison approach, we also included two applications of random-regression best linear unbiased prediction (RR-BLUP) model [20, 37], as it is one of the most widely-used statistical models in GS. The first is a univariate application from the rrBLUP R package [38], in which a separate RR-BLUP model was fitted to each trait. The second is a multivariate Bayesian application from the BGLR package called Bayesian ridge regression (BRR) [39], where a single model was fitted to all 20 traits in maize and again to all six traits in sorghum. To evaluate the ability of each of these models to accurately predict GEBVs, we conducted 50 replicates of 5-fold cross validation [CV; see [40], for a general description of the application of CV to GS studies]. For each replicate of 5-fold cross validation, the data were randomly split into training and test data, with 80% of the data used as the training set and 20% used as the test set. For each training set, the six evaluated models were fitted to data, and the GEBVs were predicted for the individuals in the corresponding test set. We note that this 5-fold cross validation procedure is equivalent to the CV1 cross-validation procedure described previously for multi-environment genomic prediction (e.g., in [41]), in that all traits are masked in the test set. Notably, similarly to [40, 41] we use CV as a sampling scheme to assess the prediction accuracy of all six comparison approaches on unobserved data, not for the purpose of selecting regularization parameters. For the four multi-trait regularized models, we selected the “best” regularization parameters in each training set by holding out 20% of the respective training data. We then used these regularization parameters to refit these regularized models on the full training set to obtain estimates of **B**, **C**_**g**_, and **C**_**e**_. For all six evaluated models, the estimates of **B**, **C**_**g**_, and **C**_**e**_ were then used to obtain GEBVs in the test set. Prediction accuracy was then determined by calculating the Pearson correlation coefficient between the observed phenotypes in the test set and the GEBVs. Please see Additional file 1: “Appendix.CV.Algorithmic.Description” for an algorithmic description of the cross validation procedure in its entirety.

### Evaluating regularized mtLLMs for GWAS

For the GWAS task, we compared the four aforementioned multi-trait approaches to the single-trait LMM with single marker (st-LMM-sm) and multitrait LMM with single marker (mt-LMM-sm) approaches. These two models test for the association of one marker at a time, on single traits and multi-traits respectively. We ran 50 experiments, where for each experiment a subsample of 80% of the data is drawn. For each method we counted the number of times each marker was detected over the 50 experiments. For st-LMM-sm and mt-LMM-sm, a marker was considered to be detected for a subsample if its Benjamini–Hochberg(BH)-adjusted *P*-value [42] was statistically significant at a false discovery rate (FDR) of $5\%.$ For the regularized multi-trait approaches mt-LMM-L1, mt-LM-L1, mt-LMM-clust and mt-LM-clust, a marker was considered to be detected if it was estimated to be non-zero.

#### Additional GWAS experiments with simulated phenotypes

We also conducted a simulation study to quantify the advantage of regularized mt-LMM approaches in terms of the detection accuracy of quantitative trait nucleotides (QTNs) underlying simulated traits, where the ground truth is known. To make data simulation as plausible as possible, we use the actual genotype matrices and kinship matrices from the two real datasets above, and covariance matrices $\varvec{C}_g$ and $\varvec{C}_e$ estimated from our regularized approaches on these datasets. Phenotypes are simulated following the mtLMM as in equation (1). The fixed additive effects of the QTNs, denoted $\varvec{B}$, were configured into four scenarios:

1. Random Structure: The QTNs randomly impacted the various traits. Hence the pattern of QTNs were trait-specific. In this case, $B_{:, j}$ and $B_{:, j'}$ were independent for any $j\ne j'$.
2. Clean Clustering Structure: The traits were divided into three groups, and the traits within a group share a common set of QTNs. In this case, $B_{:, j}$ and $B_{:, j'}$ have the same non-zero entries if trait *j* and trait $j'$ belonged to the same group. Otherwise, $B_{:, i}$ and $B_{:, j}$ are independent.
3. Mix of shared and specific: This was a superposition of random and clean clustering structures. The matrix $\varvec{B}$ was generated by summing up case 1 and case 2.
4. No fixed effects.

In scenarios 1 and 2, a total of 100 QTNs were simulated per trait. Similarly, each trait simulated under scenario 3 considered 20 QTNs from scenario 1 and 80 from scenario 2. For scenario 1, nonzero values in $\varvec{B}$ were generated sampling from the normal distribution *N*(0, 1). For scenario 2, we considered three groups of traits: two groups of three traits each, and one group of $q-6$ traits, where *q* is the total number of traits. The non zero entries within each group were generated as $B_{ij} = \mu _{ij} + \epsilon _{ij}$ where $\epsilon _{i,j}\sim N(0,0.25)$ and $\mu _{ij}\sim \text {Uniform}[-1,1]$ to include various effect strengths and signs. For scenario 3, $\varvec{B}$ was set as $\varvec{B}^{(1)}+\varvec{B}^{(2)}$ where $\varvec{B}^{(1)}$ and $\varvec{B}^{(2)}$ were generated according to scenarios (1 and 2) respectively.

For each scenario we generated 50 replicates of traits. We then applied methods stLMM-sm, mtLMM-sm, mtLMM-L1, mtLM-L1 mtLMM-clust, mtLM-clust to every replicate.

For each replicate, a non-regularized method (st-LMM-sm and mt-LMM-sm) was said to have detected a true positive QTN if its BH-adjusted *P*-value was statistically significant at an FDR of $5\%,$ whereas it was said to have detected a false positive whenever a marker that was not a QTN in the “true” model was considered to be statistically significant.

For each replicate, a regularized method (mt-LMM-L1, mt-LM-L1, mt-LMM-clust and mt-LM-clust) was said to have detected a true positive QTN whenever a QTN was included in the estimated model, whereas it was said to have detected a false positive whenever it included a marker that was not a QTN in the “true” model.

For scenarios 1–3, we then reported the average F1 score over the 50 replicates, which is the harmonic mean between precision and recall, while for scenario 4 we reported the false positive rate.

## Results

### Predictive modeling evaluation

**Fig. 1:**
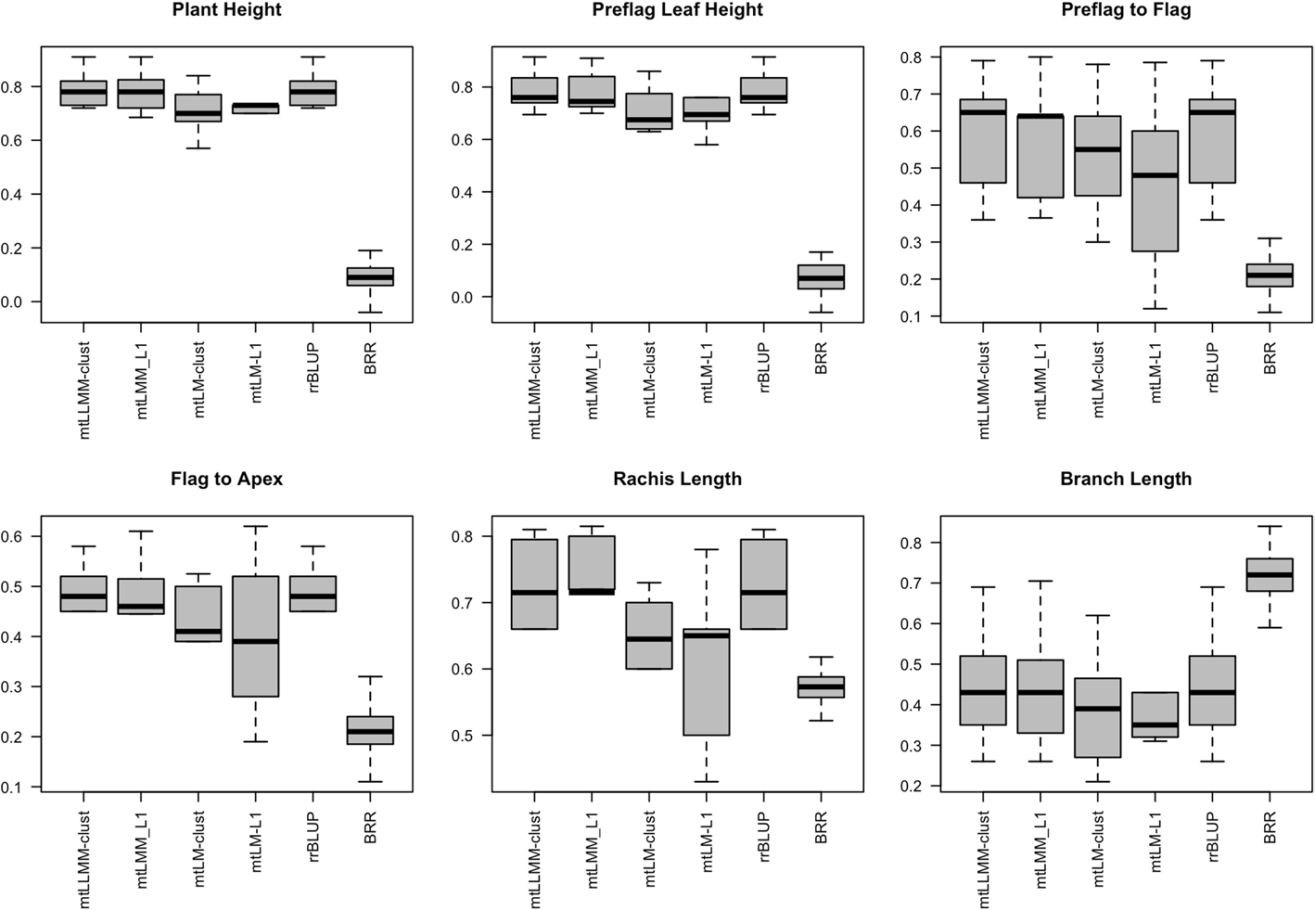
Box plots of the Pearson correlation for comparison approaches on 50 random train-test splits for the Sorghum dataset

We present the results of our evaluation of the potential of using the multi-trait regularized models for genomic prediction, using the maize and sorghum data sets. The results of the analysis of the sorghum data are presented in Fig. 1, and of the 20 tocochromanol maize grain traits in Table 1. When analyzed in sorghum, the four multi-trait regularized models (mtLMM-L1, mtLM-L1, mtLMM-clust, and mtLM-clust) performed comparably to the single-trait RR-BLUP model in sorhgum, but outperformed the multi-trait RR-BLUP model for five of the six traits. In maize, one of the multitrait regularized models, mtLMM-clust, yielded the highest mean prediction accuracies for many of the traits; however the single-trait RR-BLUP model produced the highest mean prediction accuracies for seven of the 20 traits. These results confirm the utility of regularization in conjunction with the multi-trait linear mixed model in accounting for spurious associations across crops with contrasting mating systems. In particular, regularized mtLMM approaches significantly outperformed their mtLM counterparts. We also note that approaches with clustering regularization (e.g. mtLMM-clust) led to superior results compared with their respective L1 counterparts, as they were able to take advantage of the interdependencies between the phenotypes.

**Table 1:** Average Pearson correlation and standard deviation for comparison approaches on 50 random train-test splits for the Maize dataset: multitrait linear mixed model with L1+trait-wise clustering penalty (mtLMM-clust), multitrait linear mixed model with L1 penalty (mtLMM-L1), multitrait linear model with L1+trait-wise clustering penalty (mtLM-clust),multitrait linear model with L1 penalty (mtLM-L1), single-trait RR-BLUP, applied to the rrBLUP R package (rrBLUP), multi-trait RR-BLUP, called Bayesian ridge regression (BRR) in the BGLR R package

Trait	mtLMM-clust	mtLMM-L1	mtLM-clust	mtLM-L1	rrBLUP	BRR
$\delta$T3	$0.42 \pm 0.11$	$0.39 \pm 0.08$	$0.32 \pm 0.17$	$0.28 \pm 0.14$	$\varvec{ 0.43 \pm 0.13}$	$0.23 \pm 0.06$
$\gamma$T3	$\varvec{0.56 \pm 0.13}$	$0.54 \pm 0.15$	$0.35 \pm 0.12$	$0.25 \pm 0.17$	$0.41 \pm 0.14$	$0.27 \pm 0.05$
$\alpha$T3	$0.46 \pm 0.15$	$0.44 \pm 0.25$	$0.48 \pm 0.23$	$0.44 \pm 0.14$	$\varvec{0.51 \pm 0.13}$	$0.38 \pm 0.05$
$\delta$T	$\varvec{0.48 \pm 0.17}$	$0.42 \pm 0.36$	$0.23 \pm 0.38$	$0.13 \pm 0.13$	$0.42 \pm 0.15$	$0.28 \pm 0.05$
$\gamma$T	$0.29 \pm 0.12$	$0.26 \pm 0.23$	$0.25 \pm 0.26$	$0.23 \pm 0.13$	$\varvec{0.34 \pm 0.17}$	$0.23 \pm 0.06$
$\alpha$T	$\varvec{0.68 \pm 0.14}$	$0.61 \pm 0.17$	$0.55 \pm 0.10$	$0.56 \pm 0.13$	$0.52 \pm 0.13$	$0.36 \pm 0.04$
PC8	$0.53 \pm 0.17$	$\varvec{0.54 \pm 0.27}$	$0.34 \pm 0.27$	$0.23 \pm 0.17$	$0.43 \pm 0.14$	$0.26 \pm 0.05$
Total T3	$\varvec{0.55 \pm 0.13}$	$0.52 \pm 0.19$	$0.26 \pm 0.17$	$0.25 \pm 0.15$	$0.42 \pm 0.14$	$0.32 \pm 0.05$
Total T	$\varvec{0.34 \pm 0.15}$	$0.34 \pm 0.24$	$0.28 \pm 0.29$	$0.34 \pm 0.14$	$0.30 \pm 0.17$	$0.20 \pm 0.06$
Total T3 + T	$\varvec{0.40 \pm 0.11}$	$0.36 \pm 0.12$	$0.37 \pm 0.14$	$0.27 \pm 0.17$	$0.34 \pm 0.15$	$0.23 \pm 0.05$
$\delta$T/($\gamma$T + $\alpha$T)	$\varvec{0.54 \pm 0.13}$	$0.53 \pm 0.19$	$0.27 \pm 0.23$	$0.14 \pm 0.10$	$0.44 \pm 0.15$	$0.32 \pm 0.05$
$\delta$T/$\gamma$T	$\varvec{0.48 \pm 0.14}$	$0.48 \pm 0.18$	$0.43 \pm 0.23$	$0.14 \pm 0.11$	$0.33 \pm 0.17$	$0.26 \pm 0.05$
$\delta$T/$\alpha$ T	$\varvec{0.66 \pm 0.14}$	$0.62 \pm 0.09$	$0.52 \pm 0.12$	$0.46 \pm 0.12$	$0.59 \pm 0.09$	$0.35 \pm 0.05$
$\gamma$T/($\gamma$T + $\alpha$T)	$\varvec{0.58 \pm 0.13}$	$0.55 \pm 0.14$	$0.57 \pm 0.16$	$0.48 \pm 0.12$	$0.55 \pm 0.11$	$0.33 \pm 0.06$
$\delta$T3/($\gamma$T3 + $\alpha$T3)	$0.31 \pm 0.10$	$0.33 \pm 0.14$	$0.27 \pm 0.22$	$0.34 \pm 0.14$	$\varvec{0.38 \pm 0.17}$	$0.26 \pm 0.06$
$\delta$T3/$\gamma$T3	$0.29 \pm 0.13$	$0.28 \pm 0.23$	$0.21 \pm 0.29$	$0.34 \pm 0.14$	$\varvec{0.30 \pm 0.16}$	$0.23 \pm 0.06$
$\delta$T3/$\alpha$T3	$0.38 \pm 0.09$	$0.38 \pm 0.13$	$0.25 \pm 0.14$	$0.24 \pm 0.14$	$\varvec{0.42 \pm 0.13}$	$0.26 \pm 0.05$
$\gamma$T3/($\gamma$T3 + $\alpha$T3)	$\varvec{0.45 \pm 0.13}$	$0.44 \pm 0.17$	$0.34 \pm 0.15$	$0.21 \pm 0.11$	$0.38 \pm 0.12$	$0.16 \pm 0.06$
$\alpha$T/$\gamma$T	$0.52 \pm 0.12$	$0.49 \pm 0.18$	$0.52 \pm 0.19$	$0.54 \pm 0.11$	$\varvec{0.53 \pm 0.12}$	$0.35 \pm 0.05$
$\alpha$T3/$\gamma$T3	$0.41 \pm 0.12$	$\varvec{0.43 \pm 0.16}$	$0.32 \pm 0.12$	$0.29 \pm 0.13$	$0.39 \pm 0.12$	$0.14 \pm 0.06$
All	$\varvec{0.47\pm 0.13}$	$0.45\pm 0.18$	$0.36\pm 0.20$	$0.31\pm 0.13$	$0.42\pm 0.14$	$0.27 \pm 0.05$

Values in bold-faced font indicate the model with the highest Pearson correlation

The CV1 cross validation procedure was used as described in the Materials and Methods, and performance was evaluated on unseen lines. The 20 traits, listed in the first column, considered are tocopherol (T) and tocotrienol (T3) traits measured in maize grain from [31]. $\delta$T3: delta-tocotrienol; $\gamma$T3: gamma-tocotrienol; $\alpha$T3: alpha-tocotrienol; $\delta$T: delta-tocopherol; $\gamma$T: gamma-tocopherol; $\alpha$T: alpha-tocopherol; PC8: Plastochromanol-8; Total T3: $\delta$T3 + $\gamma$T3 + *α*T3; Total T: $\delta$T + *γ*T + *α*T; Total T3  +  T: Total T3 + Total T; The remaining traits are ratios of the tocopherol or tocotrienol compounds previously described

### GWAS experiments

**Fig. 2:**
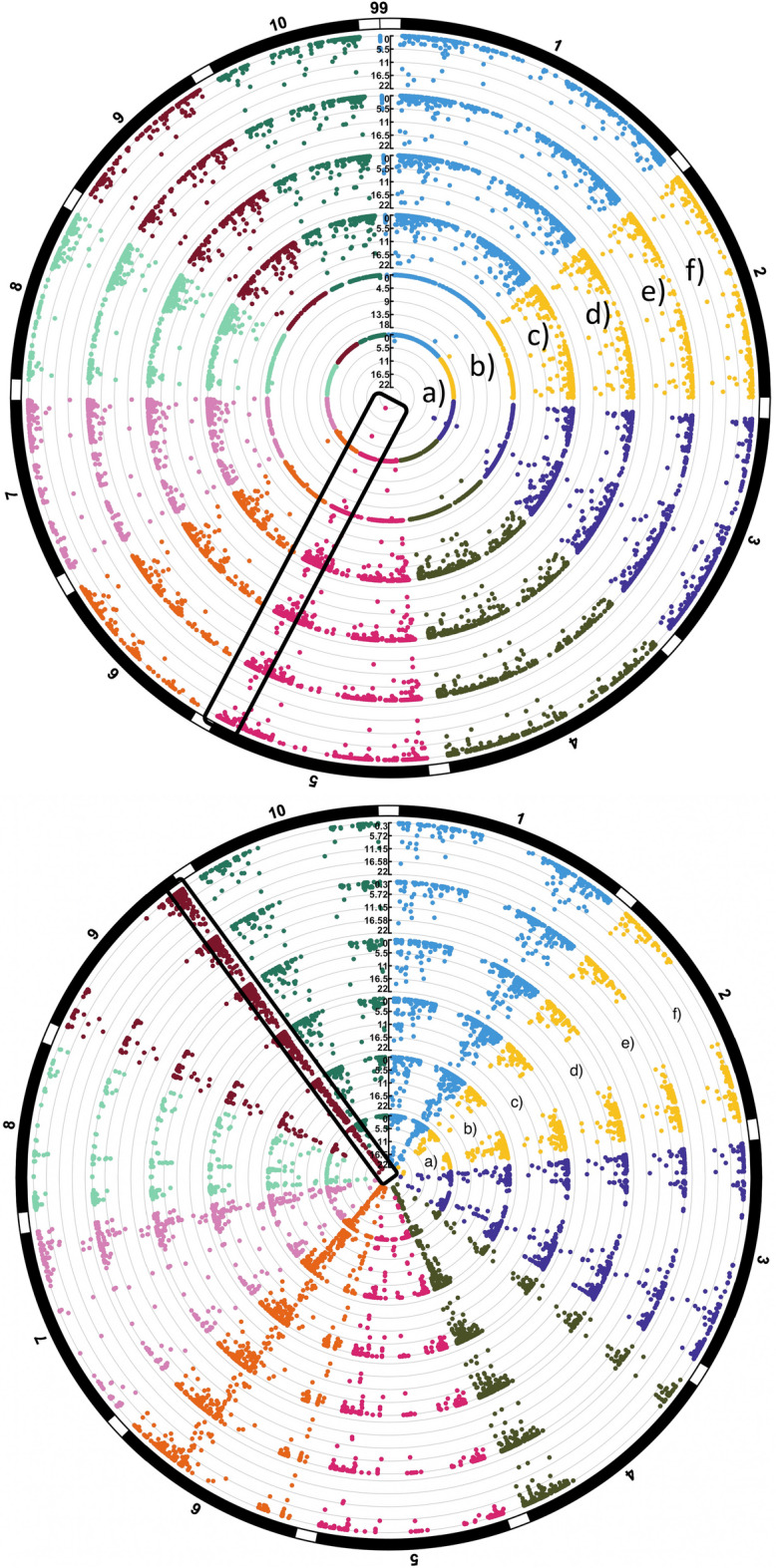
Circular Manhattan plot representing marker significance counts on 50 random $80\%$ sub-samples of top: the Maize dataset and $\alpha T$ trait; bottom: the Sorghum dataset and Plant Height trait. The X-axis depicts the physical position of markers on the reference genome, the Y-axis depicts the maximum proportion of times each marker was detected for **a** single trait linear mixed model with single marker testing (stLMM-sm), **b** multitrait linear mixed model with single marker testing (mtLMM-sm), **c** multitrait linear model with L1 penalty (mtLM-L1), **d** multitrait linear model with L1 + trait-wise clustering penalty (mtLM-clust), **e** multitrait linear mixed model with L1 penalty (mtLMM-L1), and **f** multitrait linear mixed model with L1 + trait-wise clustering penalty (mtLMM-clust)

The circular Manhattan plots presented in Fig. 2 focus on representative traits in maize and sorghum in which peak marker-trait associations have been found in previous studies. For these two representative traits, we observed that all multi-trait approaches tended to identify more peak marker-trait associations than the single-trait approaches. We also observed that the regularized models that included all markers as explanatory variables in the model tended to identify more associations than the models that only include a single marker in the model. However, this result needs to be further explored because the regularized models used a different criterion for declaring a marker to be associated with a trait. Finally, for both of the traits presented in Fig. 2, all models were able to identify associations that colocalized to regions identified in previous studies. Specifically, the GWAS presented for $\alpha$-tocopherol in maize grain identified similar peak associations in the Chromosome 5 region surrounding *ZmVTE4* to those that were published in [31], while the analysis of plant height in sorghum identified the same genomic region on Chromosome 9 region that was published in [5]. Collectively, these findings underscore the potential for regularized models, which account for spurious associations arising from population structure and familial relatedness, to identify peak GWAS associations that are consistent with previous findings.

#### GWAS simulation experiments

The F1 scores under scenarios 1–3, and false positive detection rate under scenario 4, for the various comparison approaches are provided in Tables 2 and 3, respectively. Here we observed that regularized approaches consistently outperformed single test ones in terms of detection accuracy. We also observed that regularized LMM approaches generally outperformed regularized LM methods. The results also indicate that clustering approaches can be beneficial, except for Scenario 1 in which there is no group structure among the traits and no such benefits are expected by design.

**Table 2:** Average F1 Score (the higher the better) for comparison methods on the Maize (M) and Sorghum (S) datasets, for scenarios 1,2,3

Scenario	stLMM-sm	mtLMM-sm	mtLMM-L1	mtLM-L1	mtLMM-clust	mtLM-clust
M-1	0.311	0.380	$\varvec{0.734}$	0.705	0.667	0.684
M-2	0.315	0.440	0.687	0.618	$\varvec{0.885}$	0.783
M-3	0.331	0.406	0.714	0.715	$\varvec{0.751 }$	0.746
S-1	0.348	0.356	0.426	$\varvec{0.517}$	0.513	0.427
S-2	0.345	0.388	0.423	0.315	$\varvec{0.601}$	0.594
S-3	0.341	0.383	0.415	0.445	$\varvec{0.586}$	0.384

Values in bold-faced font indicate the model with the highest average F1 score

**Table 3:** Average False Positive Rate (the lower the better) for comparison methods on the Maize (M) and Sorghum (S) datasets, for scenarios 4

Scenario	stLMM-sm	mtLMM-sm	mtLMM-L1	mtLM-L1	mtLMM-clust	mtLM-clust
M-4	$3.22 \times 10^{-3}$	$5.04 \times 10^{-4}$	$5.82 \times 10^{-4}$	$2.08 \times 10^{-3}$	$\varvec{8.70 \times 10^{-5}}$	$1.10 \times 10^{-3}$
S-4	$1.03 \times 10^{-2}$	$8.52\times 10^{-5}$	$7.71\times 10^{-5}$	$9.44\times 10^{-4}$	$\varvec{1.06\times 10^{-5}}$	$6.84\times 10^{-4}$

Values in bold-faced font indicate the model with the lowest Average False Positive Rate

## Discussion

One of the primary goals of this study was to evaluate the extent to which accounting for subpopulation structure and familial relatedness via mtLMMs improved overall performance relative to mtLMs. We also sought to determine if the mtLMM could offer additional benefits to GWAS practices through its inclusion of multiple loci and multiple traits in a single model. The results of our experiments suggest empirical confirmation of these potential benefits. In the simulated GWAS study, we clearly saw that mtLMMs provides a substantial improvement in the accuracy of marker-trait association detection, indicating in particular that it is adequately controlling for false positives. Our expectation is that the same would be true in the actual GWAS studies, wherein the added random effects in mtLMM’s would help reduce the false positive rates - this will need to be validated by plant geneticists via downstream experiments. Although this additional complexity essentially results in fitting each marker as a fixed and random effect in the model, our results suggest that the additional complexity introduced in the formulation of mtLMMs is well-motivated because of the improved model accuracy we observed. Despite this inherent additional complexity, we were able to achieve sufficient computational efficiency to merit the use of the mtLMM in data sets of similar size to the maize and sorghum diversity panels we evaluated. Collectively, these promising results suggest that the mtLMM makes it feasible to include a large number of traits in a single, unified model. Such a model would not only streamline the overall process of conducting GWAS for multiple traits, but also improve the model accuracy by leveraging structural information common across the traits.

While the ideal and ultimate test of effectiveness is an actual GWAS study where all ground truth associations are known, there are proxies that can provide partial evidence and meaningful insights. Such proxies motivated our simulation study, where we simulated traits controlled by population structure and familial relatedness in addition to the simulated QTNs. Another proxy for examining a GWAS method’s ability to control for false positives due to population structure and familial relatedness is via the “QQ-plot” of the *P*-values from the model [as in e.g., 36]. While this is a viable approach for un-regularized methods such as st-LMM-sm and mt-LMM-sm, it is not readily feasible for mtLMM due to the well-known challenge in *P*-value derivation for regularized methods. One future research direction that can investigate this limitation would be to develop easy-to-use approaches to enable unbiased estimation and hypothesis testing of the peak associations identified using regularized approaches such as those evaluated in this study.

While GWAS was the main application of this work, we also evaluated the potential for the application of the mtLMM to GS. In the ensuing analyses, the mtLMM yielded prediction accuracies that were competitive to the popular RR-BLUP model. This suggests that the use of the mtLMM for GS is reasonable, and in particular that it has comparable potential as RR-BLUP to accelerate genetic gain. Although we are not suggesting that the mtLMM be used instead of the vast amount of available GS models that are proven to be effective, the observed prediction accuracies from the mtLMM are encouraging. If the mtLMM were to be used in GS, it would then be possible to make accurate inferences on the genetic architecture of multiple traits within the training population. Excitingly, it should be relatively straightforward to incorporate the regularized approaches of the mtLMM into multi-kernel GS models (see [43] for are view of multi-kernel GS models). Thus, it could be possible to use the mtLMM in advanced GS models that can account for genotype-by-environment interactions [41], transcriptomic information [44], and potentially also in GS models similar to those in [45–47] that estimate distinct marker effects of specific effects within subpopulations.

## Conclusions

The overall positive results of the our analysis on simulated and real trait data suggest that regularized mtLMMs might be useful for both GWAS and GS. It is our hope that regularized mtLMMs will facilitate both basic agronomy-related research in plant biology and efforts to expedite crop breeding endeavors. Relevant future work directions towards these goals include (i) performing downstream biological analysis of the SNPs selected by our approaches on the real GWAS datasets, and (ii) extending the theory of statistical significance testing in the high-dimensional setting to develop a principled test for the variables selected by regularized mtLMMs.

### Supplementary Information

**Additional file 1.** An algorithmic description of the cross-validation procedure considered when evaluating prediction accuracy in the genomic selection study.

**Additional file 2.** Optimization procedure for efficiently estimating multi-trait linear mixed models (mtLMMs).

## Data Availability

The datasets analysed during the current study are available in the figshare repository at https://figshare.com/articles/dataset/Lozano_et_al_2022/20052149. These data have been previously published and are freely available to the public. The Maize Goodman diversity panel is available from [30]. The kinship matrix we employed for this panel is available from [31]. The sorghum diversity panel(*Sorghum bicolor* L. Moench) is available in [5] and [33]. The kinship matrix we used for this panel is available from [36]. A Python implementation of our methods is available along with examples at https://github.com/aclozanohuang/mtLMMreg.

## Abbreviations

GWAS: Genome-wide association studies
GS: Genomic selection
LMM: Linear mixed model
LM: Linear model
SNP: Single Nucleotide Polymorphism
mtLM: Multi-trait linear model
stLMM: Single-trait linear mixed model
stLMM-sm: Single-trait linear mixed model with single marker
mtLMM: Multi-trait linear mixed model
mtLMM-sm: Multi-trait linear mixed model with single marker
mtLMM-clust: Multi-trait linear mixed model with convex clustering regularization
mtLM-clust: Multi-trait linear model with convex clustering regularization
mtLMM-L1: Multi-trait linear mixed model with L1-norm regularization
rrBLUP: Ridge-regression Best Linear Unbiasied Predictor
QTN: Quantitative trait nucleotide
L-BFGS: Limited memory Broyden–Fletcher–Goldfarb–Shanno algorithm
