# Supplementary material for: Regularized multi-trait multi-locus linear mixed models for genome-wide association studies and genomic selection in crops
Source: BMC Bioinformatics. 2023 Oct 26;24:399. doi: 10.1186/s12859-023-05519-2 (PMC10604903; doi:10.1186/s12859-023-05519-2)
Supplement: Supplementary file 1 — Additional file 1. An algorithmic description of the cross-validation procedure considered when evaluating prediction accuracy in the genomic selection study. [file 12859_2023_5519_MOESM1_ESM.pdf]

Appendix: Cross Validation Procedure for Genomic Selection

The overall 5-fold cross validation procedure proceeds as follows.

Randomly split the data into 5 groups of equal size.
for $i = 1, \ldots, 5$ **do**
  Set the ith group as test set (20% of the full dataset) and the remaing groups as training set (80% of the full data).
  if *the model is a multi-trait regularized model* **then**
    Hold out 20% of the training set.
    Fit the model on the rest of the training set for various regularization parameters choices.
    Pick the "best" regularization parameters, i.e., the choice with highest prediction accuracy on the holdout set, where the prediction accuracy of a given choice of regularization parameters is the Pearson correlation coefficient between the observed phenotypes in the holdout set and the GEBVs obtained using the estimated B, C_g and C_e for that choice .
    Refit the model with the "best" choice on the full training set to obtain estimates of B, C_g and C_e.
  end
  else
    Fit the model on the training set to obtain estimates of B, C_g and C_e.
  end
  Use the estimates of B,C_g and C_e to obtain GEBVs in the test set.
  Compute the prediction accuracy by calculating the Pearson correlation coefficient between the observed phenotypes in the test set and the GEBVs.
end

Algorithm 1: Description of the 5-Fold Cross-Validation Procedure

In our experiments, the above procedure is repeated 50 times.

Author details

References
