# Supplementary material for: Regularized multi-trait multi-locus linear mixed models for genome-wide association studies and genomic selection in crops
Source: BMC Bioinformatics. 2023 Oct 26;24:399. doi: 10.1186/s12859-023-05519-2 (PMC10604903; doi:10.1186/s12859-023-05519-2)
Supplement: Supplementary file 2 — Additional file 2. Optimization procedure for efficiently estimating multi-trait linear mixed models (mtLMMs). [file 12859_2023_5519_MOESM2_ESM.pdf]

Appendix: Optimization for estimating regularized mtLMMs

The overall optimization proceeds as follows.

1. Optimize for $\boldsymbol{C}_g$ and $\boldsymbol{C}_e$ fixing $\boldsymbol{B}$ by employing L-BFGS[1] [1, see details below].
2. Optimize for $\boldsymbol{B}$ fixing $\boldsymbol{C}_g$ and $\boldsymbol{C}_e$ by employing proximal optimization (see details below).

Each step does not need to be run to completion. Partial updates can be performed instead.

Optimizing for $\boldsymbol{C}_g$ and $\boldsymbol{C}_e$. Fixing $\boldsymbol{B}$, let $\boldsymbol{U} = \boldsymbol{Y} - \boldsymbol{X}\boldsymbol{B}$. Let $\boldsymbol{A} = \boldsymbol{C}_g \otimes \boldsymbol{K} + \boldsymbol{C}_e \otimes \boldsymbol{I}_n$. Then optimizing for $\boldsymbol{C}_g$ and $\boldsymbol{C}_e$ amounts to minimizing

$$\frac{1}{2}\log\det(\boldsymbol{A}) - \frac{1}{2}\mathrm{vec}(\boldsymbol{U})'\boldsymbol{A}^{-1}\mathrm{vec}(\boldsymbol{U}).$$

This can be accomplished very efficiently by using the L-BFGS algorithm [1]. As described in [2], using tools from linear algebra, computing A^{-1} can be calculated in $O(n^3 + q^3)$ time where the $O(n^3)$ operation needs to be done only once in the whole analysis. Evaluating $\log\det(\boldsymbol{A})$ takes $O(nq)$. Evaluating $\mathrm{vec}(\boldsymbol{U})'\boldsymbol{A}^{-1}\mathrm{vec}(\boldsymbol{U})$ takes $O(n^2q + nq^2)$ where the $O(n^2q)$ operation is done only once prior to the analysis. Evaluating the gradient of the objective can be done in $O(n^3 + q^3)$ operations where the $O(n^3)$ operation is computed only once.

If q is too large, we employ LiMMBo [3] which randomly selects b subsets of s traits from the full set of q traits. For each subset, the variance decomposition is estimated via L-BFGS as described above. For each bootstrap, the $s \times s$ covariance matrices C_{sg} and C_{se} are combined to form the full C_g and C_e matrices as follows. First, the covariance estimates for each trait pair are averaged over the number of times they were drawn. The covariance estimates of the b subsets are then combined by a least-squares fit to the closest positive-semidefinite matrices. This reduced the overall complexity to $O(n^2 + bt_1(ns^4 + s^5) + t_2d^2)$, where t_1 and t_2 are the numbers of iterations for the two step optimization described above.

Optimizing for $\boldsymbol{B}$. Fixing $\boldsymbol{C}_g$ and $\boldsymbol{C}_e$ and defining the matrix $\boldsymbol{A} = \boldsymbol{C}_g \otimes \boldsymbol{K} + \boldsymbol{C}_e \otimes \boldsymbol{I}_n$, optimizing for $\boldsymbol{B}$ boils down to solving:

[1] L-BFGS refers to the "Limited memory Broyden-Fletcher-Goldfarb-Shanno" algorithm.

$$\underset{\boldsymbol{B}}{\text{minimize}} \frac{1}{2}(\text{vec}(\boldsymbol{Y} - \boldsymbol{X}\boldsymbol{B})'\boldsymbol{A}^{-1}(\text{vec}(\boldsymbol{Y} - \boldsymbol{X}\boldsymbol{B})) + \mathcal{R}(\boldsymbol{B})$$

Example 1: Variable Selection. When $\mathcal{R}(\boldsymbol{B}) = \lambda \sum_{i,j} |B_{ij}|$, this minimization is conducted by employing proximal gradient descent [4]. Let

$$g(\boldsymbol{B}) = \frac{1}{2}(\text{vec}(\boldsymbol{Y} - \boldsymbol{X}\boldsymbol{B})'\boldsymbol{A}^{-1}(\text{vec}(\boldsymbol{Y} - \boldsymbol{X}\boldsymbol{B})).$$

Then the updates for $\boldsymbol{B}$ is

$$\text{vec}(B)^{+} = \text{prox}_{\mathcal{R}(\boldsymbol{B}),t}(\text{vec}(B) - t\nabla g((B)),$$

where $t > 0$ is the step size and $\text{prox}_{\mathcal{R}(\boldsymbol{B}),t}$ boils down to the elementwise soft-thresholding of $\text{vec}(\boldsymbol{B})$ parameterized by λt [see 4]. For faster convergence one can apply an accelerated version, namely the fast iterative shrinkage-thresholding algorithm [FISTA, 5]. The per-iteration complexity of the approach is $O(pq\min(q,n))$.

Example 2: Variable Selection + Trait-wise Clustering.

When

$$\mathcal{R} = \lambda \sum_{i,j} |B_{ij}| + \gamma \sum_{(j,j')\in E} c_{jj'} \|B_{:j} - B_{:j'}\|$$

the problem can be solved via smoothing proximal gradient descent [SPG; 6]. Briefly, SPG, replaces the fusion penalty

$$\gamma \sum_{(j,j')\in E} c_{jj'} \|B_{:j} - B_{:j'}\|$$

by its smooth approximation, and then solves the resulting optimization problem using FISTA [5]. The per-iteration complexity of the approach is $O(pq\min(q,n) + q|E|)$ where $|E|$ is the number of trait pairs considered for fusion.

Notice the significant gain in complexity with respect to the number of SNPs: Prior methods could not accommodate $p >> n$ SNPs, and even if they did, their complexity would be cubic in p at best.

Author details

References

1. Liu, D.C., Nocedal, J.: On the limited memory bfgs method for large scale optimization. Mathematical programming **45**(1-3), 503–528 (1989)
2. Furlotte, N.A., Eskin, E.: Efficient multiple-trait association and estimation of genetic correlation using the matrix-variate linear mixed model. Genetics **200**(1), 59–68 (2015)
3. Meyer, H.V., Casale, F.P., Stegle, O., Birney, E.: Limmbo: a simple, scalable approach for linear mixed models in high-dimensional genetic association studies. BioRxiv, 255497 (2018)
4. Parikh, N., Boyd, S.: Proximal algorithms. Foundations and Trends in optimization **1**(3), 127–239 (2014)
5. Beck, A., Teboulle, M.: A fast iterative shrinkage-thresholding algorithm for linear inverse problems. SIAM journal on imaging sciences **2**(1), 183–202 (2009)
6. Chen, X., Lin, Q., Kim, S., Carbonell, J.G., Xing, E.P., *et al.*: Smoothing proximal gradient method for general structured sparse regression. The Annals of Applied Statistics **6**(2), 719–752 (2012)
